# Effect of sub-micron grains and defect-dipole interactions on dielectric properties of iron, cobalt, and copper doped barium titanate ceramics

**DOI:** 10.3389/fchem.2023.1249968

**Published:** 2023-09-14

**Authors:** Sara C. Mills, Eric A. Patterson, Margo L. Staruch

**Affiliations:** ^1^ U.S. Naval Research Laboratory, Materials Science and Technology Division, Washington, DC, United States; ^2^ American Society for Engineering Education (ASEE), Washington, DC, United States

**Keywords:** barium titanate, doping, ferroelectric, grain size, ceramic processing

## Abstract

**Introduction:** Dilutely doped ferroelectric materials are of interest, as engineering these materials by introducing point defects via doping often leads to unique behavior not otherwise achievable in the undoped material. For example, B-site doping with transition metals in barium titanate (BaTiO_3_, or BTO) creates defect dipoles via oxygen vacancies leading enhanced polarization, strain, and the ability to tune dielectric properties. Though defect dipoles should lead to dielectric property enhancements, the effect of grain size in polycrystalline ferroelectrics such as BTO plays a significant role in those properties as well.

**Methods:** Herein, doped BTO with 1.0% copper (Cu), iron (Fe), or cobalt (Co) was synthesized using traditional solid-state processing to observe the contribution of both defect-dipole formation and grain size on the ferroelectric and dielectric properties.

**Results and discussion:** 1.0% Cu doped BTO showed the highest polarization and strain (9.3 μC/cm^2^ and 0.1%, respectively) of the three doped BTO samples. While some results, such as the aforementioned electrical properties of the 1.0% Cu doped BTO can be explained by the strong chemical driving force of the Cu atoms to form defect dipoles with oxygen vacancies and copper’s consistent +2 valency leading to stable defect-dipole formation (versus the readily mixed valency states of Fe and Co at +2/+3), other properties cannot. For instance, all three T_c_ values should fall below that of undoped BTO (typically 120°C–135°C), but the T_c_ of 1.0% Cu BTO actually exceeds that range (139.4°C). Data presented on the average grain size and distribution of grain sizes provides insight allowing us to decouple the effect of defect dipoles and the effect of grain size on properties such as T_c_, where the 1.0% Cu BTO was shown to possess the largest overall grains, leading to its increase in T_c_.

**Conclusion/future work:** Overall, the 1% Cu BTO possessed the highest polarization, strain, and T_c_ and is a promising dopant for engineering the performance of the material. This work emphasizes the challenge of extricating one effect (such as defect-dipole formation) from another (grain size modification) inherent to doping polycrystalline BTO.

## 1 Introduction

Engineering ferroelectric ceramics by purposefully introducing point defects has a profound effect on the material’s behavior, such as unusual ferroelectric properties and high electric field induced strain (Zhao et al., 2019). Examples of point defects include dopant or impurity ions as well as vacancies, which can lead to defect dipoles arising from oxygen vacancies and ionized acceptors (Ren, 2004; Maier et al., 2015). For perovskites (ABO_3_ structure), dilute doping at the B site leads to a distribution of oxygen vacancies to achieve charge neutrality, which form defect complexes with the doped acceptor ion (Vani and Kumar, 2012; Maier et al., 2015). The oxygen vacancies have an intrinsic mobility, which is affected by the dopant itself, the concentration of dopant, and the transition metal’s valence state. Barium titanate (BaTiO_3_, BTO) is a ubiquitous ferroelectric oxide with low dielectric loss, chemical stability, and is lead-free with low toxicity, and has been widely and commonly doped at the Ti^4+^ site with various transition metals, including iron (Fe), manganese (Mn), cobalt (Co), and copper (Cu) (Rani et al., 2016). Although there are a multitude of studies on such dopants, these works focus on dopant concentrations that lead to hexagonal BTO, do not include polarization/strain curves in addition to permittivity and loss measurements, study ferromagnetic or optical properties, are processed under different thermal profiles, and do not attempt to directly compare three different dopants in the same work (Langhammer et al., 2003; Cheng et al., 2005; Dang et al., 2011; Deka et al., 2014; Langhammer et al., 2015; Langhammer et al., 2016; Rani et al., 2016; Krimech and Sayouri, 2020; Althobaiti, 2023).

In perovskites, such as dilutely doped BTO, both the ionic radius and valency of the dopant is highly important in determining the site of doping (either A or B site). This is closely related to how effective the particular dopant will be at trapping oxygen vacancies, which is highly influenced by the valence state and the charge neutrality conditions of the composition (Eichel, 2008; Schie et al., 2014). In addition, the concentration of oxygen vacancies is influenced by the concentration and specific element chosen as the dopant (Huang et al., 2014). B-site dopants with a lower valency than Ti^4+^ (i.e. 2+ or 3+) can then form stable defect dipoles with oxygen vacancies along the <001> direction, the same direction as the spontaneous polarization direction (Zhao et al., 2019). These particular defect-dipole complexes have been shown to have a profound effect on the ferroelectric and piezoelectric behavior of materials such as barium titanate. The moment of the defect dipole induces an internal bias field, which can increase the polarization of the material by producing large crystal field anisotropy in the vicinity of the dopant and, when poled in the direction of the defect dipole, can also increase the strain experienced in the material (Maier et al., 2015). The orientation of the dipoles takes place during oxygen vacancy diffusion on the scale of several unit cells, which occurs at room temperature and leads to a recoverable domain wall configuration and “pinched” hysteresis loops with decreased remnant polarization (Kamel and de With, 2008; Zhao et al., 2019). Overall, a dopant cation has a direct effect over the range of several unit cells on the association and migration energies, i.e., the energies of oxygen vacancy—dopant association and activation energy of vacancy motion away from the dopant, respectively (Schie et al., 2014). This can significantly impact the formation of these complexes and thus the bulk ferroelectric properties.

Although our previous work (Patterson et al., 2023) and several other works discussed above focus on single crystals, for polycrystalline samples another important influence on the ferroelectric and piezoelectric properties of both doped and undoped BTO is grain size. It has been reported that several electrical properties such as the piezoelectric constant (d_33_), polarization, and relative permittivity are affected by grain size, typically with a decrease in these properties with a decrease in grain size as grains approach the nanometer scale (Zhao et al., 2004; Guo et al., 2012; Huan et al., 2014). This is predicted to be due to a change in domain density and non-uniformities at grain boundaries (Huan et al., 2014). It has also been observed that non-uniformities such as aluminum segregation at grain boundaries can be partially responsible for ferroelectric aging behavior even when no second phase is detected, separate from impacts of volume of domain walls (Carl and Hardtl, 1977). Overall, domain configuration has a large impact on macroscopic dielectric properties, which in turn is substantially affected by the microstructure (Eichel, 2008). However, it is imperative to attempt to separate the effects of microstructure and intrinsic effects on the dielectric properties of polycrystalline ferroelectrics.

In this work, dielectric materials properties will be investigated (polarization, strain, and relative permittivity/loss) and reported with respect to polycrystalline BTO ceramics doped with 1.0 mol% of Fe, Co or Cu, synthesized via traditional solid-state processing. These results will then be discussed with respect to the grain size, determined from cross-sectional scanning electron microscopy (SEM) images. Comparisons between the dielectric properties of the three different doped BTO samples show the importance of considering all of the various contributions from the dopant that can affect the dielectric properties. These can be due to defect-dipole formation, grain growth/growth inhibition, oxygen vacancy diffusion, or a complex contribution from a subset or all of these. The challenge of extricating one effect from the other will be emphasized, along with the need for these in-depth studies.

## 2 Materials and methods

Ceramic powders of BaTi_1-x_Me_x_O_3_ (with Me = Fe, Co, or Cu) were prepared using traditional solid state synthesis methods with x = 0.01 mol. Precursor oxides of BaCO_3_, TiO_2_, Fe_2_O_3_, CoCO_3_, and CuO (99.9+%) were mixed in ethanol and ball milled for 8 h with proper stoichiometric amounts of the Fe, Co or Cu precursor added. The mixed powders were dried and then calcined at 1,150°C for 2 h and milled and dried again as before. Pellets of each composition were then uniaxially pressed in a 12.7 mm diameter die after mixing the calcined powder with 4-5 wt% binder (Paraloid B-72 resin mixed with acetone). The pressed pellets were sintered at 1,250°C in air for 4 h. The density of the sintered pellets was greater than 91% theoretical density for all samples in this study. The phase of the pellets was confirmed with X-ray diffraction (XRD) using a Rigaku SmartLab Rigaku X-ray powder diffractometer with Cu Kα radiation at 40 kV and 200 mA for a 2θ range from 20° to 80° with a 0.02° step size.

The sintered pellets were then ground down to a thickness of approximately 800 µm and masked and sputtered with gold electrodes for synchronous ferroelectric and piezoelectric measurements, which were performed with a self-constructed Sawyer-Tower system and a linear variable differential transformer (LVDT) connected to a lock-in amplifier. The electrical measurements were performed on samples in three different states with increasing applied voltages from 0.1 kV up to 1.4 kV at 1 Hz (all data shown in this work is at 1.4 kV) as the properties are well-understood to strongly depend on the defect configuration, which can be modified through temperature or electric field. Therefore, the thermal and electrical history of the sample are important factors in discussing the dielectric behavior. The three states to be measured are referred hereafter as: as-sintered, aged, and poled. All samples were thermally reset at 300°C for 4 h after the initial sintering and allowed to cool to room temperature. This default “as-sintered” state was then tested. The “aged” subset of these samples were then aged at 80°C for 24 h to allow for diffusion of oxygen vacancies prior to characterization. Finally, the “poled” subset of samples were poled at a DC bias of 1.5 kV for 1 h at room temperature and tested.

Simultaneous capacitance and tan δ measurements were performed using two different methods in order to determine the relative permittivity and dielectric loss of the samples under different conditions. All samples for these measurements were tested in the “as-sintered state.” First, an Agilent E4980A precision LCR meter was used to sweep between frequencies of 200 Hz and 2 MHz to determine the permittivity and loss at room temperature. Second, a Thermal Product Solutions (TPS) Tenney Environmental Test Chamber was used to measure the capacitance and loss from −30°C to 155°C at four different frequencies: 0.1, 1, 10, and 100 kHz. The frequency was generated from an HP 4284A Precision LCR meter, which also measured the capacitance and loss at each frequency over the temperature range. A self-assembled LCR test interface was used to connect the LCR meter to the sample holder inside the environmental test chamber, which was accompanied by a National Instruments thermocouple placed close to the sample to more accurately monitor temperature.

Cross-sectional scanning electron microscopy (SEM) images were taken of the polished, fractured surface of each composition using a Thermo Scientific Quattro S at an accelerating voltage of 10 keV. Images were taken across different regions of the cross-section, and were analyzed by ImageJ software. Ten images were used to determine the average grain size via the linear intercept method, whereby five lines were drawn per image to obtain an average grain size for one image, resulting in 50 total lines drawn with ten average values of grain size for each particular sample.

## 3 Results and discussion

### 3.1 X-ray diffraction

**FIGURE 1:**
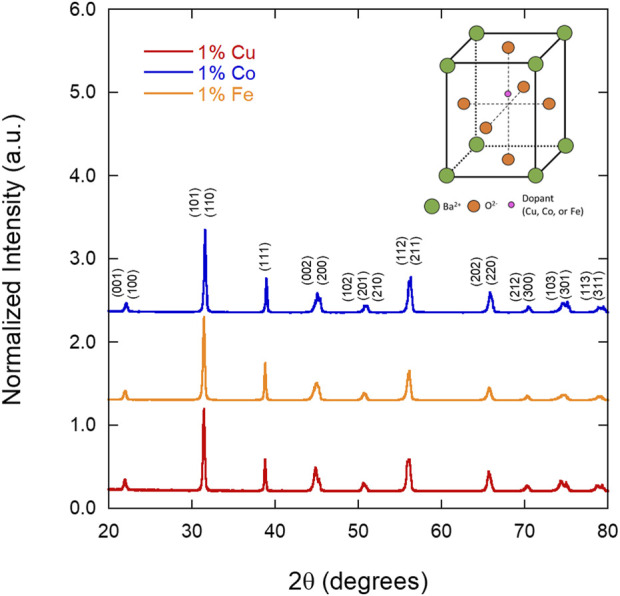
X-ray diffraction patterns of the 1% Fe, Co, and Cu-doped BTO powders measured after calcination at 1150°C, showing the tetragonal phase of BTO and no evidence of a hexagonal phase impurity (indexed with respect to PDF# 04-012-8129). The crystal structure of tetragonal BTO is shown in the top right corner.

Sintered pellets of 1% Fe, Co, and Cu-doped BTO were analyzed via XRD to determine if the concentration of Fe affected the presence of the expected ferroelectric, tetragonal phase, as some studies have observed the formation of the hexagonal phase at as low as 1% Fe (Langhammer et al., 2020). Figure 1 shows that across the three samples, the calcined powders are of the ferroelectric tetragonal phase with no other phases present, demonstrating that doping in this study with Fe, Co, or Cu does not induce a detectable phase change at x = 1%. The lattice parameters and c/a ratios obtained from a whole pattern fit of each diffraction pattern (indexed with respect to tetragonal BTO, PDF# 04-012-8129) is included in Table 1. The c/a ratio of undoped BTO, 1.01, is slightly higher than that of the Co, Fe, and Cu-doped BTO samples (1.008, 1.007, and 1.008, respectively). Doping with 1% Co and Fe has been shown to slightly decrease the c/a ratio, which was observed here for all three dopants (Buscaglia et al., 2000).

**TABLE 1:** Lattice parameters, dielectric properties, average grain sizes, and densities for the three doped BTO samples.

	Lattice parameters			@ 10 kHz					
	a,b	c	c/a	ε_r_	tan δ	$T_{c_{H}}$ (°C)	$T_{c_{C}}$ (°C)	Avg. grain size (µm)	Relative density (%)
1% Co BTO	4.002	4.033	1.008	1830	0.017	107.1	90.2	0.62 ± 0.2	95.2
								2.29 ± 0.3	
1% Fe BTO	4.005	4.033	1.007	2240	0.012	99.8	61.2	0.88 ± 0.2	96.9
								49.19 ± 13.5	
1% Cu BTO	4.004	4.036	1.008	2150	0.026	139.4	103.6	4.73 ± 1.1	91.4
								14.35 ± 4.4	
								32.57 ± 3.8	

Dielectric properties are reported for samples measured at 10 kHz, and the Curie temperature is reported both on heating ( $T_{c_{H}}$ ) and on cooling ( $T_{c_{C}}$ ). Lattice parameters were obtained from a whole pattern fit of the XRD patterns of each sample. Grain size was measured using the linear-intercept method. Density was measured using the Archimedes method.

### 3.2 Ferroelectric and piezoelectric testing

**FIGURE 2:**
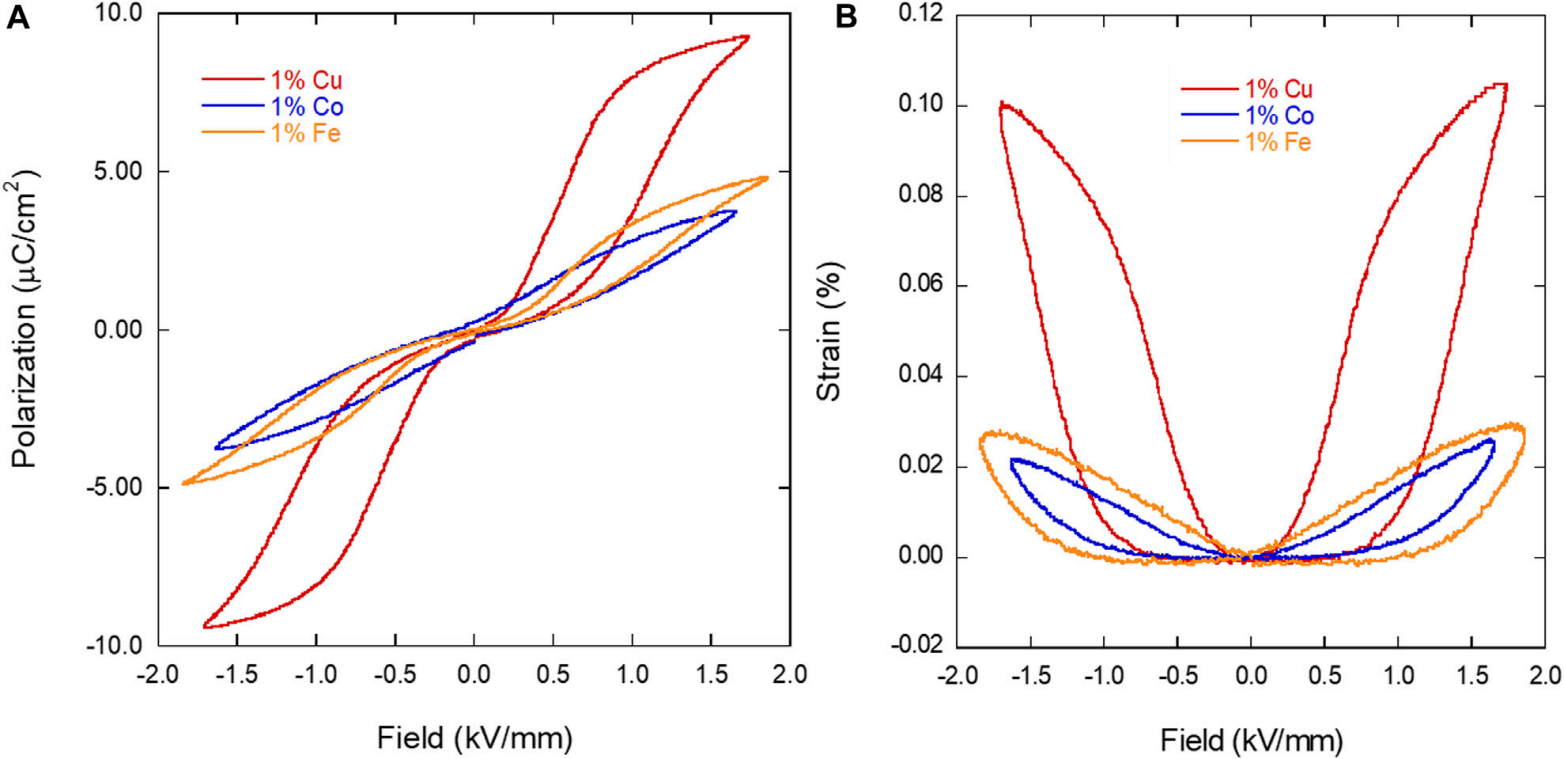
Polarization **(A)** and strain **(B)** loops for the thermally reset (300°C) 1% Fe, Co and Cu-doped BTO samples measured at 1.4 kV.

Polarization and strain of the three compositions in all three conditions were measured at 1.4 kV, which translates to a field of approximately 1.5 kV/mm. Figure 2 shows polarization (Figure 2A) and strain loops (Figure 2B) for the “as-sintered” condition measured at 1.4 kV. Note that all samples were tested at applied voltages between 0.1 and 1.4 kV at 1 Hz, but only the 1.4 kV results are shown here. The polarization and strain loops for the samples that were aged (80°C, 24 h) and poled (1.5 kV, 1 h at 2.4 kV) can be found in the Supplementary Material (Supplementary Figures S1, S2). Measuring samples after aging and poling was performed to determine the extent that temperature and field cycling affects the polarization and strain of these differently doped materials. The 1.0% Fe-doped BTO sample showed an increase in strain from ∼0.025% to ∼0.04% after aging, and the 1.0% Cu BTO sample showed a decrease in polarization from ∼9 to ∼5 μC/cm^2^ (Supplementary Figure S1). In order to fully realize the effect on the polarization and strain after poling, the poled samples were measured at 2.4 kV (Supplementary Figure S2), showing multiple changes in polarization and strain behaviour. It is important to not compare their maximum values without considering that the field level is nearly twice as high as in the as-sintered and aged state. With that in mind, the 1.0% Cu BTO sample displayed no further enhancement compared to the “as-sintered” value of polarization and the pinched portion of the polarization hysteresis loop disappears in favor of a traditional ferroelectric shape. The strain also takes on a more butterfly shape with negative strain at the coercive field. Additionally, the polarization loops of the 1.0% Co and Fe samples have become asymmetric, indicating a high internal electric field (Supplementary Figure S2A). This is supported by the offset in the field of the 1.0% Co and Fe BTO strain loops (Supplementary Figure S2B). The poled strain loops also show a general improvement of strain for all three samples that approaches 0.1%, albeit at higher fields as mentioned. Aging and poling can have extreme effects on the polarization and strain behaviour of ferroelectric materials via domain/defect-dipole orientations (Kamel and de With, 2008). While the effects that both aging or poling have on the polarization and strain of the material via an external, post-processing method are quite large, in these samples they were found to be repeatably reversible following a 300°C heat treatment. Importantly, the hysteresis loops always returned to the “as-sintered” state, so it is from this baseline condition that comparisons will be focused on in this discussion.

In the “as-sintered” state, the 1.0% Cu-doped BTO yields the highest polarization (∼9.3 μC/cm^2^) and strain (∼0.1%) when compared to the 1.0% Fe and Co-doped BTO, with polarizations of 4.8 and 3.7 μC/cm^2^ and strains of 0.03% and 0.025%, respectively. In comparison, undoped BTO sintered at similar conditions yield polarizations between 15 and 18 μC/cm^2^ (Tan et al., 2015; Malyshkina et al., 2020). Additionally, Figure 2B shows that all three dopants yielded little to no negative strain, showing recoverable strain across each dopant. The presence of the pinched polarization loops of the 1.0% Fe and Cu-doped BTO samples (Figure 2A) is a good indication of defect dipole formation pinning domain walls in those samples.

There is an obvious improvement in the ferroelectric properties with respect to the 1.0% Cu-doped BTO when compared to the 1.0% Fe and Co. Looking at each dopant, however, it does not seem at first glance that ionic radius plays a part, as the difference between the ionic radii for the various oxidation states of each transition metal are relatively similar when compared to Ti^4+^ (0.61) (listed in Å): 0.75 (Co^2+^), 0.55 (Co^3+^), 0.77 (Cu^+^), 0.73 (Cu^2+^), 0.78 (Fe^2+^), and 0.65 (Fe^3+^) (Shannon, 1976). Therefore, doping with any of these three elements for Ti^4+^ should not play a role in one composition more than another. It is important to note, however, that the valence state of the incorporated ion is dependent on a number of factors, such as initial precursors and the oxidizing/reducing conditions during synthesis. Therefore, we should expect a mix of these valence states for some if not all of the dopants chosen in this work.

While the valence states of the incorporated dopants will not significantly distort the lattice, the valency of the ions can influence the intrinsic ferroelectric behaviour. Co and Fe are both stable at a 2+ or 3+ state, while Cu is commonly found in a 2+ state. The mixed valence of the Co and Fe may play a role in achieving charge neutrality (via different amounts of oxygen vacancies and other free charges) which then affects the ferroelectric and piezoelectric properties of the material. This difference in valence is proposed to directly affect the establishment of defect-dipoles, formed between the dopant and induced oxygen vacancies. Using Kröger-Vink notation, the defect-dipole complexes for the three different dopants are the following: ${\left({Fe}_{Ti}^{\prime }-V_{o}^{∙∙}\right)}^{∙}$ , ${\left({Co}_{Ti}^{\prime }-V_{o}^{∙∙}\right)}^{∙}$ , and ${\left({Cu}_{Ti}^{\prime \prime }-V_{o}^{∙∙}\right)}^{X}$ . The charge states of the three different defect dipole complexes are based on the assumption that Fe and Co are both in a 3+ state and Cu is 2+. This was assumed because it has been shown that, for Fe and Co, oxidation from the 2+ to 3+ state is more energetically favourable in comparison to the incorporation of those two elements in their divalent state (Buscaglia et al., 2001). Due to the charged nature of the defect-dipole complexes for Fe and Co in the 3+ state, there are additional mechanisms for charge compensation that may take place, including free electrons trapped in the lattice (Eichel, 2008). This excess charge can give rise to a screening effect that ultimately causes ferroelectric instability, which could explain the decreased polarization and strain in the 1% Fe and Co-BTO samples when compared to the 1% Cu sample (Wang et al., 2012). Additionally, work by Erhart et al. (2007) suggests that there is a very strong chemical driving force between copper dopants and oxygen vacancies in lead titanate, which theoretically leads to all copper ions complexed with an oxygen vacancy.

### 3.3 Other dielectric measurements

#### 3.3.1 Relative permittivity (ε_r_)/tan δ vs. frequency (room temperature)

**FIGURE 3:**
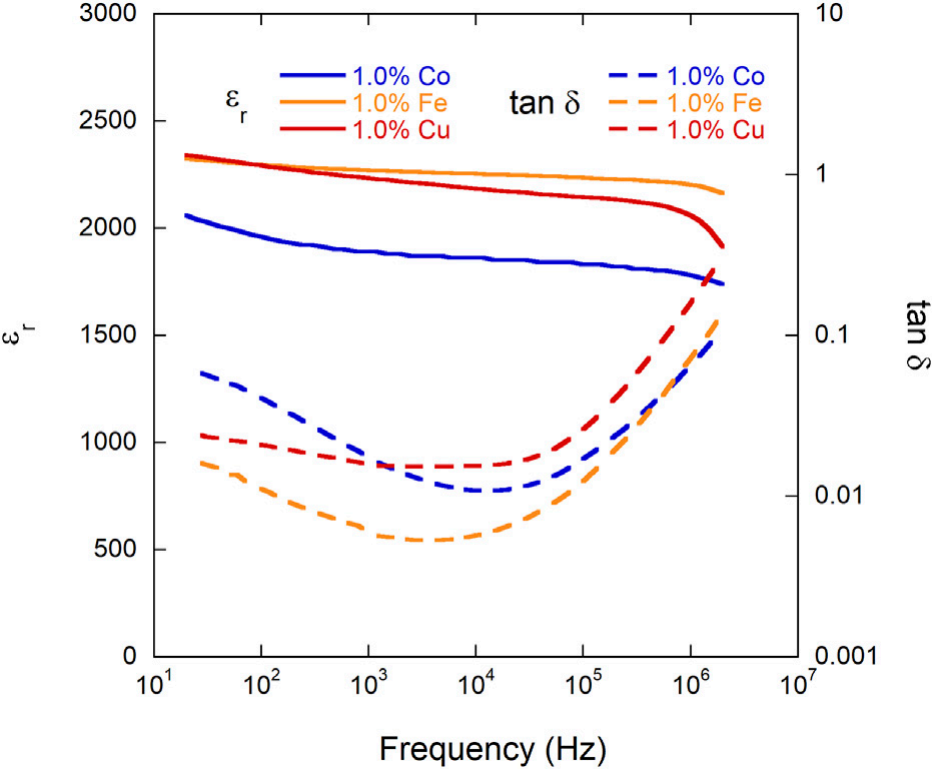
Relative permittivity (ε_r_) (solid lines) and loss tangent (tan δ) (dashed lines) of each composition (1% Co, Cu, and Fe BTO) measured at room temperature with respect to frequency after samples were thermally reset at 300°C for 4 h.

The relative permittivity (ε_r_) and loss tangent (tan δ) of as-sintered samples of each composition were measured at room temperature with respect to frequency, shown in Figure 3 and reported in Table 1. When compared to the typical relative permittivity of barium titanate (ε_r_∼3,000-6,000), all three of the doped BTO samples exhibit a lower value than undoped BTO, which is due to the fact that in comparison to Ti^4+^, all three dopants (Co^3+^, Cu^2+^, and Fe^3+^) are more conductive and less polarizable, decreasing the relative permittivity, as expected (Rani et al., 2016). More specifically, undoped BTO at similar processing conditions yields a relative permittivity similar to the reported ranged of BTO, ranging from ∼3,000 to 6,500 at 1 kHz (Hoshina et al., 2008; Tan et al., 2015). However, the permittivity of all three doped BTO samples remains fairly constant up to 2 MHz, due to the failure of the dipoles to follow the fast alternating electric field (Rani et al., 2016). With regards to tan δ at 10 kHz, each doped sample exhibited a loss of ∼0.01, a relatively low value of loss due to the dopant’s ability to reduce leakage current in the sample (Imai et al., 2002). All samples showed an increase in loss in the MHz, frequency range and the 1% Cu doped BTO sample nominally yielded the highest loss at 2 MHz. The Cu doped sample, however, showed the most frequency-stable loss at the intermediate to lower frequencies (with the increase starting at 100 kHz).

#### 3.3.2 Relative permittivity (ε_r_)/tan δ vs. temperature (variable frequency)

**FIGURE 4:**
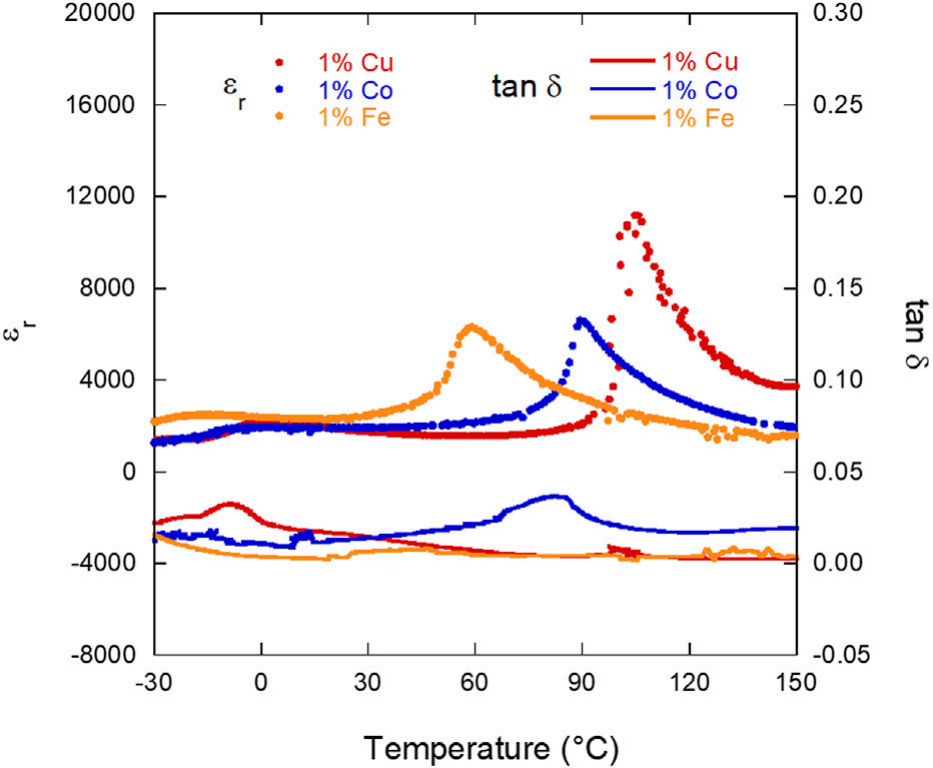
Relative permittivity (ε_r_) (solid lines) and loss tangent (tan δ) (dashed lines) of each composition (1% Co, Cu, and Fe BTO) with respect to temperature, measured at 10 kHz in the “as-sintered” state (thermally reset at 300°C for 4 h after sintering). Only the cooling curves are shown in this plot.

As-sintered samples of 1% Co, Cu, and Fe doped BTO were loaded into an LCR test interface and the ε_r_ and tan δ were measured between −30°C and 155°C at 0.1, 1, 10, and 100 kHz. Data from the cooling curves for each sample at 10 kHz are shown in Figure 4 with Curie temperatures measured both on heating ( $T_{c_{H}}$ ) and cooling ( $T_{c_{C}}$ ) are reported in Table 1. Plots of ε_r_ and tan δ with respect to temperature at all four frequencies are included in the Supplementary Material (Supplementary Figure S3). The magnitude of ε_r_ is highest at the Curie temperature for 1% Cu (∼7,200) and is similar for 1% Co and 1% Fe (∼5,520). Additionally, the $T_{c_{H}}$ of the 1.0% Cu-doped BTO (139.4°C) is higher than both the 1.0% Co (107.1°C) and 1.0% Fe (99.8°C) samples. One can also observe that there is a slight increase in tan δ at $T_{c_{H}}$ for all three dopants, and that the loss is consistently highest in the 1% Co sample. To further explore the differences in T_c_ values of the three dopants, the difference between the T_c_ on heating and cooling (ΔT_Ch-Cc_) have been calculated as 16.9, 38.6, and 35.8°C for Co, Fe, and Cu doped BTO, respectively. The smaller ΔT_Ch-Cc_ of the 1% Co and the similar ΔT_Ch-Cc_ values of the 1% Fe and Cu doped BTO samples could be due to the relative abilities of each dopant to trap vacancies in the sample, which would lead either more or less thermal energy needed to complete the phase transition between the tetragonal and cubic phases.

With respect to the $T_{c_{H}}$ of the three samples, it is important to note that the accepted value of T_c_ for undoped BTO lies between 120°C and 135°C, with samples processed under comparable conditions yielding Curie temperatures ranging between 120°C and 130°C (Tan et al., 2015; Malyshkina et al., 2020). It is generally expected that doping BTO with these cations or ones of similar valency will decrease the T_c_ due to oxygen vacancy formation (Tewatia et al., 2021). The 1.0% Co and 1.0% Fe samples follow this trend in lower $T_{c_{H}}$ than the reported value of undoped BTO: 1.0% Co (107.1°C) and 1.0% Fe (99.8°C). The similarity in $T_{c_{H}}$ of the 1.0% Co and Fe samples follows the similarity of their trends in other properties, such as polarization and strain, which could be due to their similarities in valence (3+), which would lead to similar defect-dipole formation and subsequent effects on ferroelectric properties as a result. In contrast, the $T_{c_{H}}$ of 1.0% Cu BTO was 139.4°C, higher than the maximum of the accepted range of T_c_ of undoped BTO. This suggests that there may be another factor at play that is driving the $T_{c_{H}}$ higher, as the introduction of oxygen vacancies should decrease the T_c_. One such factor is the grain size in the material. Properties such as polarization and relative permittivity are affected by grain size, with a decrease in these properties as grains approach the nanometer scale.

### 3.4 Grain size measurement

**FIGURE 5:**
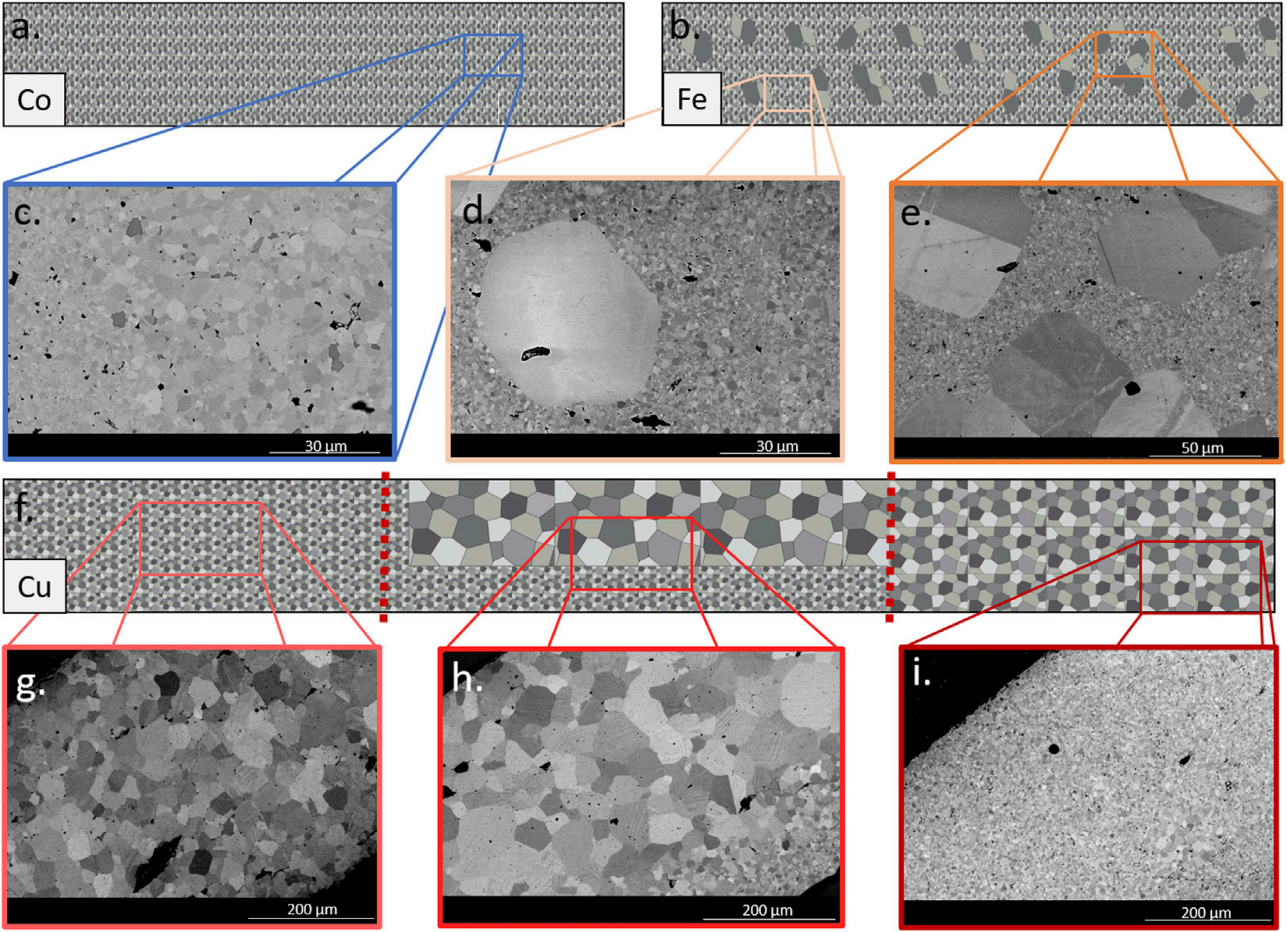
Schematics illustrating the grain structure of cross sections of 1.0% Co BTO **(A)**, 1.0% Fe BTO **(B)** and 1.0% Cu BTO **(F)** samples. Magnified areas are SEM images from each sample [1.0% Co **(C)**, 1.0% Fe **(D,E)**, and 1.0% Cu **(G–I)**] showing the grain structure and size distribution along the length of the cross section.

In order to measure the grain sizes of the three samples, cross-sections of a pellet of each dopant were polished and then imaged via SEM. Grain size was then determined by the linear intercept method, utilizing ten SEM images to obtain average grain sizes for the different grain size distributions within each sample. Schematics of each sample illustrating the grain structure of each sample can be seen in Figures 5A, B, F for Co, Fe and Cu dopants, respectively. Micrographs from different sub-sections of these representative cross-section schematics are also included in Figures 5C–E, G–I. The average grain size values can be found in Table 1.

The cross-section of the 1.0% Co sample (Figures 5A, C) had a minor but distinctly bimodal distribution of grains (0.62 ± 0.2 and 2.29 ± 0.3 µm), revealing a population of submicron grains. These two groups of grains were equally distributed throughout the cross-section of the sample, as illustrated in Figure 5A. Next, the 1.0% Fe sample (schematically shown in Figure 5B) also had a bimodal distribution of grains (0.88 ± 0.2 and 49.19 ± 13.5 µm), but here the subset of larger grains were substantially bigger compared to the Co sample. For clarity, it is important to note that the larger grains of this sample were measured individually (measuring 175 grains to obtain the average), while the smaller grains were measured by the linear intercept method. Unlike 1.0% Co BTO, 1.0% Fe BTO had clusters of these large grains surrounded by the smaller, submicron grains, shown in the SEM images in Figures 5D, E, which are representative of what was observed along the entirety of the cross-section. Finally, the 1.0% Cu sample (illustrated in Figure 5F) had three distinct areas along the cross-section (Figures 5G–I) and three different populations of grain sizes (4.73 ± 1.1, 14.35 ± 4.4, and 32.57 ± 3.8 µm). Overall, 1.0% Cu had the largest grains of the three different dopants, albeit not distributed evenly over the cross-section. Upon electron dispersive spectroscopy (EDS) analysis, there was a lack of apparent dopant segregation into grain boundaries or secondary phases in the cross-sections of the doped samples. Undoped BTO sintered under similar conditions have grains averaging approximately 4 µm in diameter, the size of which is very dependent on the sintering temperature and time (Hoshina et al., 2008; Tan et al., 2015; Malyshkina et al., 2020). The choice of dopant itself is likely driving the observed differences in grain sizes between the three samples, since they were sintered intentionally under the same conditions to achieve similar high density (>91%) rather than optimizing the sintering conditions to create a specific grain size. In previous doping studies, for instance, Co has been used to control grain sizes in ceramics to ∼1 µm by inhibiting grain growth, and Cu was added as a sintering aid to encourage grain growth (Cheng et al., 2005; Mahmoudi et al., 2019).

This grain size analysis, then, can potentially inform some of the differences in dielectric properties between the three samples. Although doping should theoretically decrease the Curie temperature, this was not observed in the 1.0% Cu sample. This increase in T_c_ could then be contributed, in part, to the larger average grain size, as it has been shown that T_c_ increases as grain size increases (Tewatia et al., 2021). Additionally, although there are larger grains present in 1.0% Fe BTO, the dominant small grains of this sample and that of the 1.0% Co sample could explain the lower T_c_, polarization, and strain values when compared to 1.0% Cu BTO. The permittivity values do not follow this trend, as the 1.0% Cu and Fe samples have similar values, while the 1.0% Co BTO possesses the lowest ε_r_. However, since these values are not vastly different (Table 1) and do fall below that of undoped BTO (ranging from 3,000 to 6,000), it may not be a significant indicator of the dependence of this value on either dopant or grain size alone. This analysis, though, does highlight the importance of considering both the defect dipole and grain size effects on dielectric properties. Although some dopants are typically classified as more or less effective than others, such as if a dopant’s valency and/or quantity added should lead to positive enhancements via defect dipole formation, the effect of grain size can outweigh the benefit of the defect dipoles with respect to dielectric properties.

## 4 Conclusion

In this work, we have studied the dielectric properties of polycrystalline B-site doped BTO with 1.0% copper (Cu), iron (Fe), and cobalt (Co), synthesized using traditional solid-state processing, to observe the contribution of both defect-dipole formation and grain size on the ferroelectric and dielectric properties. 1.0% Cu doped BTO showed the highest polarization and strain (9.3 µC/cm^2^ and 0.1%, respectively) of the three dopants in and a very clearly pinched hysteresis and recoverable strain behavior in the as-sintered state, with some changes in polarization and strain to all three doped samples after aging or poling. Additionally, 1.0% Cu BTO showed the highest Curie temperature (Tc) (139.4°C). With respect to defect dipole formation, these results suggested that the strong chemical driving force of the Cu atoms to form defect dipoles with charge neutrality induced oxygen vacancies leads to improved polarization and strain. However, since this would not explain the high Tc of the 1.0% Cu; the average grain size and distribution of grain sizes provided insight for this parameter instead. The 1.0% Cu BTO possessed the largest overall grains, which leads to an increased Tc. Grain size cannot be the only contributor to the improvement of properties, though, as the larger grain size would not account for the type of pinching in the polarization loop and increased strain, which we attribute to defect-dipole formation. Overall, this work emphasizes the challenge attributing a singular contribution, such as defect dipole formation via doping or grain size to dielectric properties of polycrystalline, doped BTO. Instead, it is critical to obtain a more comprehensive view of the material as a whole in order to inform causation and conclusions.

## Data Availability

The raw data supporting the conclusion of this article will be made available by the authors, without undue reservation.
