# Supplementary material for: Effect of sub-micron grains and defect-dipole interactions on dielectric properties of iron, cobalt, and copper doped barium titanate ceramics
Source: Front Chem. 2023 Sep 14;11:1249968. doi: 10.3389/fchem.2023.1249968 (PMC10537944; doi:10.3389/fchem.2023.1249968)
Supplement: Supplementary file 1 [file DataSheet1.docx]

Supplementary Material

Effect of sub-micron grains and defect-dipole interactions on dielectric properties of iron, cobalt, and copper doped barium titanate ceramics

Sara C. Mills^1,2^, Eric A. Patterson^1^, Margo L. Staruch^1*^

^1^U.S. Naval Research Laboratory, Materials Science and Technology Division, Washington, D.C. 20375, United States

^2^Postdoctoral Associate/Contractor, American Society for Engineering Education (ASEE), Washington, D.C. 20036, United States

*** Correspondence:**Sara C. Mills

sara.mills@nrl.navy.mil


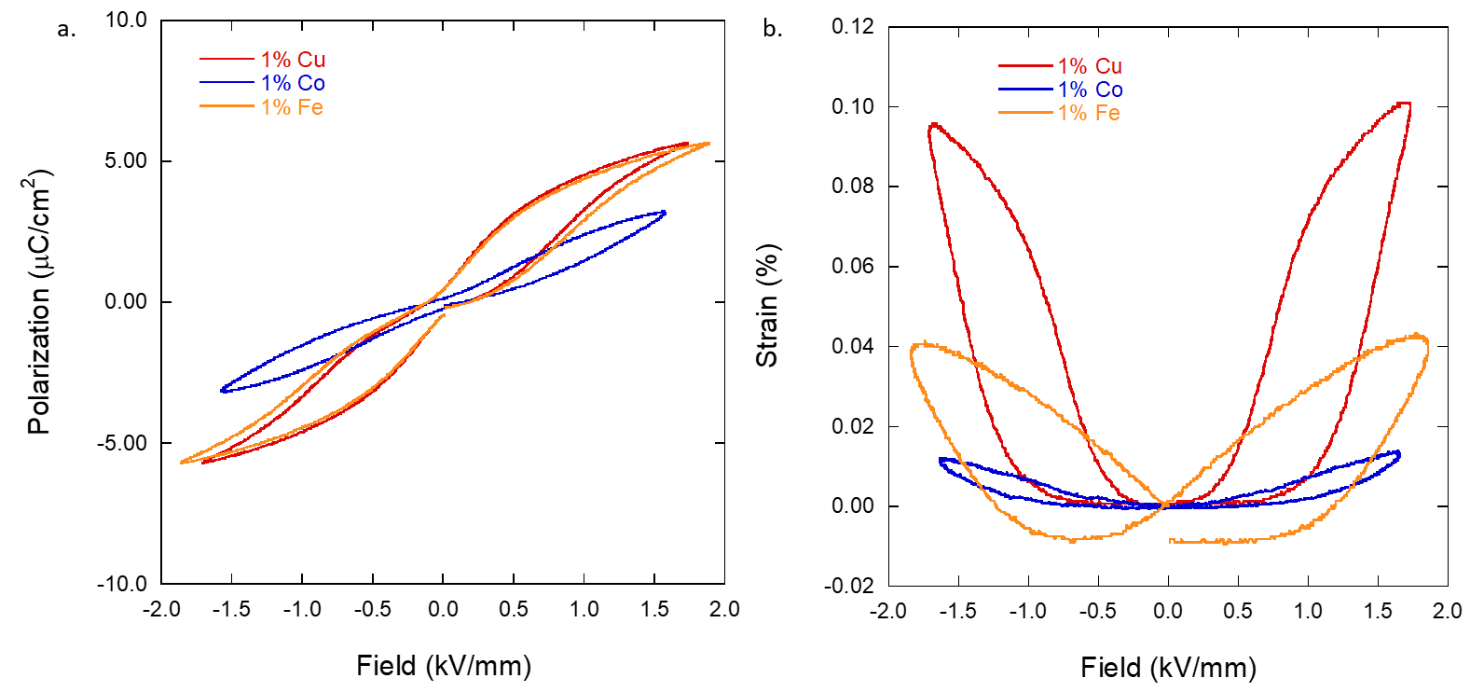


**Supplementary Figure 1.** Polarization (a) and strain (b) loops for the aged (80°C, 24 hours) 1% Fe, Co and Cu-doped BTO samples measured at 1.4 kV.

**
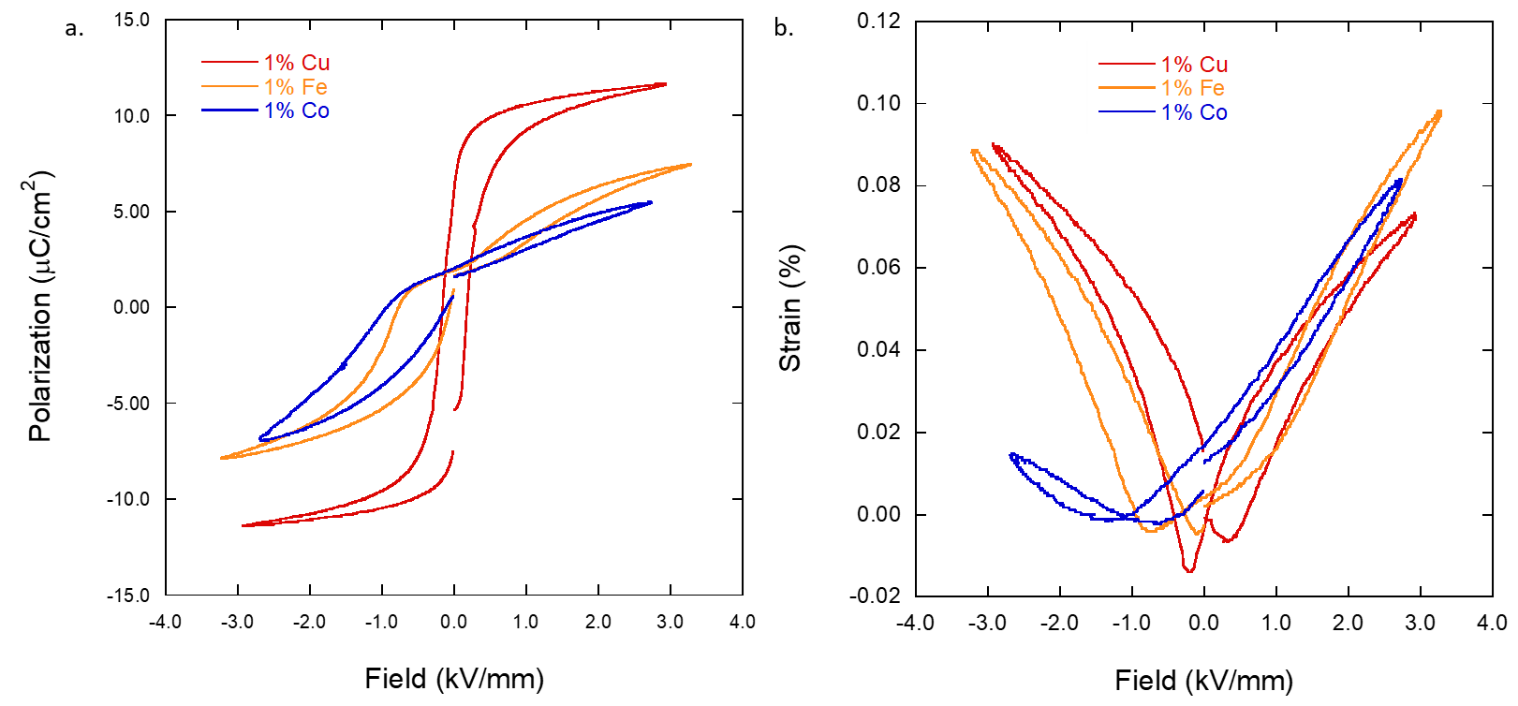
**

**Supplementary Figure 2.** Polarization (a) and strain (b) loops for the poled (1.5 kV, 1 hour) 1% Fe, Co and Cu-doped BTO samples measured at 2.4 kV.


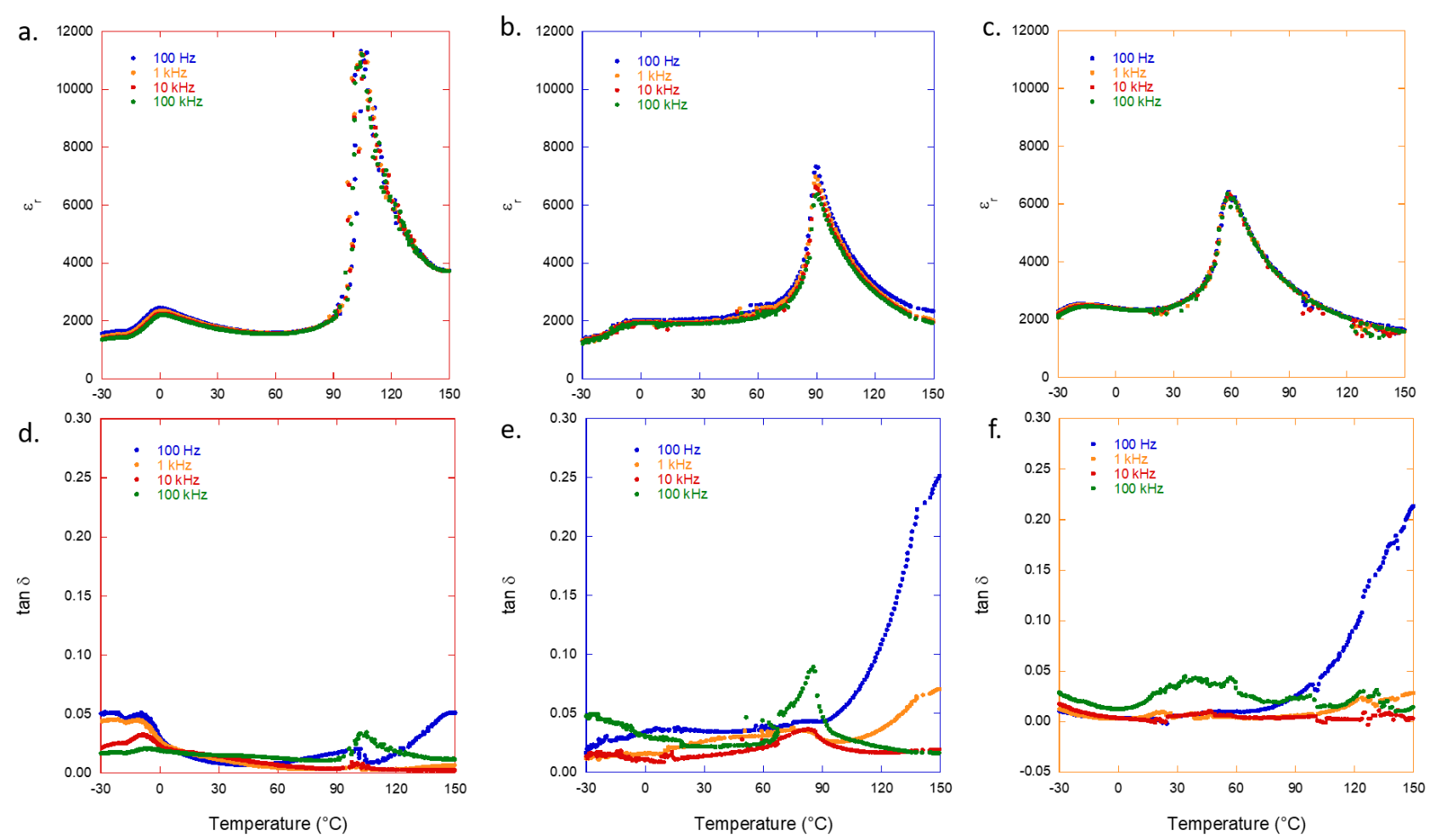


Cu

Cu

Co

Co

Fe

Fe

**Supplementary Figure 3.** Dielectric constant (ε_r_) (a-c) and loss tangent (tan δ) (d-f) of each composition (1% Cu, Co and Fe BTO) in the “as-sintered” state (thermally reset at 300°C for four hours after sintering) with respect to temperature measured at 100 Hz, 1, 10 and 100 kHz. Only the cooling curves are shown here.
